# MicroRNAs enrichment in GWAS of complex human phenotypes

**DOI:** 10.1186/s12864-015-1513-5

**Published:** 2015-04-16

**Authors:** Luiz F Goulart, Francesco Bettella, Ida E Sønderby, Andrew J Schork, Wesley K Thompson, Morten Mattingsdal, Vidar M Steen, Verena Zuber, Yunpeng Wang, Anders M Dale, PRACTICAL/ELLIPSE consortium, Ole A Andreassen, Srdjan Djurovic

**Affiliations:** NORMENT, KG Jebsen Centre for Psychosis Research, Institute of Clinical Medicine, University of Oslo, Oslo, Norway; Department of Medical Genetics, Oslo University Hospital, Oslo, Norway; Division of Mental Health and Addiction, Oslo University Hospital, 0407 Oslo, Norway; Multimodal Imaging Laboratory, University of California at San Diego, La Jolla, CA 92037 USA; Cognitive Sciences Graduate Program, University of California at San Diego, La Jolla, CA 92037 USA; Center for Human Development, University of California at San Diego, La Jolla, CA 92037 USA; Department of Psychiatry, University of California at San Diego, La Jolla, CA 92037 USA; NORMENT, KG Jebsen Centre for Psychosis Research, Department of Clinical Science, University of Bergen, Bergen, Norway; Dr. Einar Martens Research Group for Biological Psychiatry, Center for Medical Genetics and Molecular Medicine, Haukeland University Hospital, Bergen, Norway; Centre for Molecular Medicine Norway, Nordic EMBL Partnership, University of Oslo and Oslo University Hospital, Oslo, Norway; Department of Neuroscience, University of California at San Diego, La Jolla, CA 92037 USA; Department of Radiology, University of California at San Diego, La Jolla, CA 92037 USA

**Keywords:** Polygenic, Genomic enrichment, Genome-wide association study, Conditional false discovery rate, miRNA

## Abstract

**Background:**

The genotype information carried by Genome-wide association studies (GWAS) seems to have the potential to explain more of the ‘missing heritability’ of complex human phenotypes, given improved statistical approaches. Several lines of evidence support the involvement of microRNA (miRNA) and other non-coding RNA in complex human traits and diseases.

We employed a novel, genetic annotation-informed enrichment method for GWAS that captures more polygenic effects than standard GWAS analysis, to investigate if miRNA-tagging Single Nucleotide Polymorphisms (SNPs) are enriched of associations with 15 complex human phenotypes. We then leveraged the enrichment using a conditional False Discovery Rate (condFDR) approach to assess any improvement in the detection of individual miRNA SNPs associated with the disorders.

**Results:**

We found SNPs tagging miRNA transcription regions to be significantly enriched of associations with 10 of 15 phenotypes. The enrichment remained significant after controlling for affiliation to other genomic categories, and was confirmed by replication. Albeit only nominally significant, enrichment was found also in miRNA binding sites for 10 phenotypes out of 15. Leveraging the enrichment in the condFDR framework, we observed a 2-4-fold increase in discovery of SNPs tagging miRNA regions.

**Conclusions:**

Our results suggest that miRNAs play an important role in the polygenic architecture of complex human disorders and traits, and therefore that miRNAs are a genomic category that can and should be used to improve gene discovery.

**Electronic supplementary material:**

The online version of this article (doi:10.1186/s12864-015-1513-5) contains supplementary material, which is available to authorized users.

## Background

Genome-wide association studies (GWAS) have identified thousands of single nucleotide polymorphisms (SNPs) associated with complex human traits and diseases in the last years. However, these variants explain only a small proportion of the estimated heritability. The failure to explain a substantial proportion of the heritability of complex phenotypes is often described as the "missing heritability" problem [1,2]. Some of the missing heritability may lie with very rare variants of large effect yet to be detected [3] but heritable complex diseases and traits are also widely believed to have an underlying polygenic architecture [4,5] characterized by a large number of not so rare variants with effects too small to be captured by standard statistical approaches.

Recent studies have shown that the genotype information in GWASs can be used to explain more of the heritability of common complex traits than revealed by standard analytical methods [6,7]. New statistical approaches have been developed to improve SNP discovery and uncover more polygenic effects without increasing the sample size [7-9]. These new methods exploit the notion that some SNP categories are more likely to have a true effect than others. Schork et al. [7] showed that SNPs in some genomic regions (e.g. 5’UTR, 3’UTR, exons or introns) are ”enriched” of significant associations as compared to the baseline. It is a plausible assumption that other genomic categories too could be enriched of such associations with complex human phenotypes.

MicroRNAs (miRNAs) are a class of small non-coding RNAs (~22 nucleotides long) that regulate gene expression by inhibiting protein translation or by degrading the mRNA transcript [10,11]. A single miRNA may regulate hundreds of functional targets within one cell type simultaneously, providing the cell with an intricate mechanism for fine-tuning the regulation of gene expression. Over 1,500 human miRNAs are reported in miRBase version 19 (http://mirbase.org). Like protein-coding genes, miRNAs are transcribed mainly by polymerase II as long primary transcripts (pri-miRNAs) in the nucleus. However, in contrast to protein-coding genes, they are subsequently cleaved to produce stem loop structured precursor molecules (pre-miRNAs) by the nuclear RNase III enzyme Drosha along with other factors [12]. The pre-miRNAs are then exported to the cytoplasm by exportin-5 [12], where the RNase III enzyme Dicer further processes them into mature, short miRNAs that are subsequently incorporated into the RNA-induced silencing complex.

MiRNAs are able to impact a variety of cellular pathways and functions. Several studies have shown that miRNAs are critical to development, apoptosis, cell proliferation, immunity, and patterning of the nervous system [13,14]. Deregulation of miRNAs leads to a variety of human diseases [15]. Several studies suggest that around 30% to 50% of all human genes are regulated by miRNAs [11,16]. Moreover, as has emerged from GWAS data [17], more than 90% of disease-associated SNPs are located in non-coding regions of the genome, for example in promoter regions, enhancers, silencers, or even in non-coding RNA genes. It is therefore reasonable to assume that a number of such non-coding variants may affect miRNA transcription or be involved in the disruption or the creation of miRNA binding sites. Variants in miRNA primary transcripts can affect the expression of miRNAs by altering miRNA processing inside the nucleus. Variants in mature miRNAs or miRNA binding sites can generate gain and loss of function, leading to alterations of the genetic pathways in which those miRNAs are involved [17]. We therefore expect SNPs located in miRNA transcription regions and miRNA binding sites to be more likely to show significant associations with complex human phenotypes compared to baseline SNPs (“enrichment of miRNA vs. baseline SNPs”).

In order to test this hypothesis, we applied the genomic annotation-informed enrichment method first employed by Schork et al. [7]. We first calculated the relative enrichment of miRNA tagging SNPs using the summary statistics GWAS data of 15 complex human phenotypes. We then leveraged the enrichment in a conditional False Discovery Rate (condFDR) framework to assess the improvement in the detection of individual miRNA-related SNPs associated with these phenotypes. We found that SNPs tagging miRNA sites and binding sites were more likely to be associated with the phenotypes compared to baseline SNPs (i.e. intergenic SNPs), though less compared for instance to 5’UTR SNPs. A similar enrichment pattern was shown across the majority of human phenotypes investigated.

## Methods

### Samples

Genome-wide association study (GWAS) summary statistics data from 15 phenotypes including i) brain-related disorders and traits: bipolar disorder (BD) [18], schizophrenia (SCZ) [19], smoking behaviour as measured by cigarettes per day (CPD) [20], ii) cardiovascular risk factors (including metabolic diseases): systolic blood pressure (SBP) [21], plasma lipids (triglycerides, TG, high density lipoprotein, HDL, low density lipoprotein, LDL) [22], type 2 diabetes [23] iii) immune-mediated diseases: multiple sclerosis (MS) [24],Crohn’s disease (CD) [25], ulcerative colitis (UC) [26], and iv) anthropomorphic measures: body mass index (BMI) [27], height [6] waist to hip ratio (WHR) [28], and v) prostate cancer [29] were included in the present study. The summary statistics (p-values or z-scores) were obtained from public access websites or published supplementary material. A description of the underlying GWAS data is presented with Additional file 1. In total, these studies comprised approximately 1.3 million phenotypic observations, but considerable sample overlap makes the number of unique individuals lower. The p-values from the respective GWAS meta-analyses, derived according to best practices, underwent no further processing with the exception of a correction for inflation. The baseline statistic for the control of inflation was computed from intergenic SNPs [7]. Also, SNPs with reference (rs) numbers that did not map onto the 1000 genomes project (1KGP) reference panel were excluded.

### miRNAs transcription regions (miRNA)

The human miRNA data was downloaded from miRBase (www.mirbase.org) version 19, which includes published miRNA sequences and annotations. The human dataset of miRBase version 19 has 1,595 primary transcripts with genomic coordinates, name and accession number of each miRNA [30]. Before converting the miRBase human data set to a suitable file format, the primary transcript coordinate intervals of the miRNAs were extended by ten kilobases in both directions in order to include SNPs in the regulatory regions of the miRNAs themselves [31].

### miRNA binding sites (miRNA-BS)

miRNA validated targets were downloaded from miRecords (http://www.hsls.pitt.edu/obrc/index.php?page=URL1237998207), version 4, last updated April 2013. MiRecords consists of validated and predicted miRNA targets [32]. The validated targets component is a large, high-quality database of experimentally validated miRNA targets resulting from manual curation of literature. We obtained 1,214 human miRNA targets from miRecords that were used to create the miRNA target regions annotation category (see Positional annotation categories below).

### Positional annotation categories

Bi-allelic SNP genotypes from the European reference sample provided by the November 2010 release of Phase 1 of the 1KGP were obtained in pre-processed form from http://www.sph.umich.edu/csg/abecasis/MACH/download/. Basic quality control functionalities of Plink version 1.07 [33] (32) were applied: SNPs with a minor allele frequency of less than 1%, missing in more than 5% of individuals and/or violating Hardy-Weinberg equilibrium (p < 1×10^−6^) were excluded from the reference panel; individuals missing more than 10% of the genotypes were excluded. All remaining 1KGP SNPs were assigned single, mutually exclusive genic annotation categories based on their genomic position (hg19). Genic annotation categories were: 1) miRNA transcription regions (miRNA); 2) miRNA binding sites (miRNA-BS); 3) 3’-untranslated region (3’UTR); 4) intron; 5) exon; 6) 5’-untranslated region (5’UTR); 7) Enhancer; 8) Silencer; 9) transcription factor binding site (TFBS); 10) non-coding RNA (NCRNA); and 11) intergenic.

### Linkage disequilibrium (LD) weighted SNP category scoring

The use of tag SNPs in GWAS limits the discovery of functional variants within a ”tagged” linkage block, potentially overseeing the effect of other variants in only partial linkage disequilibrium (LD) with their tag SNP. In the current study the LD between SNPs was incorporated as part of a strategy devised to obtain a stronger and more consistent differentiation of enrichment among several annotation categories [7]. In our enrichment analysis an LD-weighted scoring algorithm allows the quantification of the properties of multi-locus LD structure implicitly captured by each tag SNP. The resulting LD-informed categories are leveraged as strata for the subsequent analysis [7-9].

For each GWAS tag SNP a pairwise correlation coefficient approximation to LD (r^2^) was calculated for all 1KGP SNPs within a 1,000,000 base pairs (1Mb) distance that passed quality control (see above). All r^2^ values < 0.2 were set to 0 and each SNP was assigned an r^2^ value of 1.0 with itself. LD-weighted annotation scores for each category were computed as the sum of LD r^2^ between the tag SNP and all 1KGP SNPs in that category (±10 kilobases in the case of miRNA, see above).

Given SNPi, its LD-weighted annotation score for an annotation category was computed as:

$$ \mathrm{L}\mathrm{D} \inf \mathrm{o}\mathrm{i} = \Sigma \mathrm{j}\ \left(\delta \mathrm{j}\ *\ \mathrm{r}\mathrm{i}\mathrm{j}2\right), $$

where rij2 is the LD r-squared between SNPi and SNPj and δj takes values of 1 or 0 depending on whether the 1KGP SNPj belongs to the annotation category or not. Each tag SNP was then “LD”-assigned to that annotation category if its LD-weighted annotation score was greater than or equal to 1.

### Intergenic SNPs

Intergenic SNPs were defined as having LD-weighted annotations scores for each of the other categories equal to zero and being in LD with no SNPs in the 1KGP reference panel located within 100,000 base pairs of a protein coding gene, within a non-coding RNA, within a transcription factor binding site or within a miRNA binding site. Those singled out in this way are expected to form a collection of non-genic SNPs not belonging to any (annotated) functional elements within the genome (including through LD) and therefore represent a collection of likely null associations [7].

### Intergenic Inflation Control

The empirical null distribution in GWAS is affected by global variance inflation due to population stratification and cryptic relatedness [15] and deflation due to the over-correction caused by the application of standard genomic control methods to polygenic traits [16]. We applied a control method leveraging only intergenic SNPs that are likely depleted of true associations. The following procedure was carried out for each phenotype: after conversion of p-values into z-scores, the genomic inflation factor [15], λ_GC_, was estimated as the median squared z-score across intergenic SNPs divided by the expected median of a chi-square distribution with one degree of freedom (the .95 quantile was used for CPD in place of the median). We used intergenic SNPs to estimate inflation because their relative depletion of associations suggests they provide a robust estimate of true null SNPs that is uncontaminated by polygenic effects. Using annotation categories in this fashion is important given concerns posed by recent GWAS [8] about the over-correction of test statistics using standard genomic control.

### Quantile-Quantile (Q-Q) Plots and Enrichment

Q-Q plots are used to compare two distributions. The Q-Q plots reported here show -log10 nominal p-values plotted against -log10 empirical p-values for different phenotypes, for all SNPs and for each categorical subset. Leftward deflections of the observed distribution from the projected null line reflect increased tail probabilities in the distribution of the test statistic and consequently larger proportions of low p-values with respect to the one expected by chance. An increased deflection from the null line in the Q-Q plots of summary statistics conditioned on a genomic category is referred to here as “enrichment” of that genomic category [7].

### Binomial proportion test

In order to assess the statistical significance of the enrichment, the SNPs in the summary statistics data from the GWAS studies were first pruned of LD redundancy. This was done by randomly selecting ten different sets of representatives from sliding 1Mb LD (*r*^2^ > 0.2) windows. The proportions of SNPs in all given annotation categories were subsequently compared to the proportion of intergenic SNPs in the top decile using a binomial proportion test [34]. A p-value was finally computed from the median of the binomial proportion test statistics over the ten replicas.

### Enrichment estimates

As explained in detail by Schork et al. [7], an overall estimate of the categorical enrichment graphically illustrated in the Q-Q plots is the sample mean of z^2^ - 1, where the sample mean is taken over all the SNPs z-scores in the given category. Assuming the z-score distribution in a given category to be a mixture of a standard normal null distribution and a non-null distribution also symmetric around zero, the sample mean z^2^ for the SNPs over that category provides an estimate of the variance due to all null and non-null SNPs in that category. By subtracting one, we obtain a conservative estimate of the variance in effect sizes attributable to non-null SNPs alone. The miRNA enrichment estimates for all phenotypes were computed and joined with the estimates computed by Schork et al. [7] for other genic categories. All estimates where then normalized by the largest value per phenotype.

### Regression

Another way of testing whether a covariate of interest, e.g. an LD-informed genic annotation score, predicts association with a given phenotype is to regress a proxy for the association against the covariate. We regress here the logarithm of the squared association z-scores (as we are not interested in the direction of effect) against the LD-informed genic annotations scores. The inclusion of affiliation scores for the most relevant genic categories in the regression accounts for possible latent mediation effects. Different genic categories can encompass LD-blocks of very different sizes and therefore have rather different affiliation scores distributions. These could bias the enrichment significance measured through the binomial proportion test. An estimate of the SNPs total LD is included in the regression to account for this potential bias. The total LD of a SNP (TotLD) is measured as the sum of pairwise LD *r*^2^ greater or equal than 0.2 between a SNP and all 1KGP SNPs within 1,000,000 base pairs from it.

### Conditional False Discovery Rate (condFDR)

The enrichment expressed in the Q-Q plots can be directly interpreted in terms of the Bayesian False Discovery Rate (FDR) [35]. Specifically, for a given p-value cutoff, the FDR is defined as

$$ \mathrm{F}\mathrm{D}\mathrm{R}(p) = {\pi}_0{F}_0(p)\ /\mathrm{F}(p), $$

where *π*_0_ is the proportion of null SNPs, *F*_0_ is the null cumulative distribution function (cdf), and *F* is the cdf of all SNPs, both null and non-null. Under the null hypothesis, *F*_0_(p) = p is the cdf of the uniform distribution on the unit interval [0,1], so

$$ \mathrm{F}\mathrm{D}\mathrm{R}(p) = {\pi}_0p/F(p). $$

*F* is estimated by the empirical cdf *q* = *N*_*p*_/*N*, where *N*_*p*_ is the number of SNPs with p-values less than or equal to p, and *N* is the total number of SNPs. If *π*_0_ is close to one, as is likely true for most GWASs, a reasonable conservative estimate of FDR is *p*/*q*. The negative decadic logarithm of this FDR estimate is log_10_(*q*) - log_10_(*p*) which coincides with the aforementioned deflection from the null line.

The conditional False Discovery Rate (condFDR) is conservatively estimated as FDR(*p* | *x*) = *p* / *F*(*p* | *x*), where *x* is the value of a given annotation X, and *F*(*p* | *x*) is the cdf of *p* conditional on the annotation X = *x*. If SNPs are enriched for associations according to levels of X, then *F*(*p* | *x*) > *F*(*p*) for a given p and hence FDR(*p* | *x*) < FDR(*p*).

### Replication

In order to test the specificity of the condFDR method, we looked at the replication rate across independent samples. Both the data and the procedure used to compute the conditional rate of replication closely follow the ones used by Schork et al. [7]. Initially, the z-scores of each of the eight original sub-studies contributing to the CD meta-analysis were independently adjusted using intergenic inflation control. The eight sub-studies were then subdivided into two groups of four in $$ \left(\begin{array}{c}\hfill 8\hfill \\ {}\hfill 4\hfill \end{array}\right) $$ = 70 ways, the first group, *D*_k_, *k* = 1,…,70, serving as discovery group, the second, *R*_k_, *k* = 1,…,70, as replication group. Average discovery and replication z-scores were computed for all SNPs and all 70 subdivisions and multiplied by the square root (2) of the number of substudies in the group.

The average z-scores were converted to p-values using the standard normal cumulative distribution function *Φ*. Two-tailed discovery p-values were calculated as *p*_D_ = 1-*Φ*(|*Z*_D_|) + *Φ*(−|*Z*_D_|) = 2*Φ(−|*Z*_D_|), one-tailed replication p-values preserving the signs of the discovery z-scores were calculated as *p*_R_ = *Φ*(sgn(*Z*_D_) * *Z*_R_).

Separate cumulative replication rates were calculated for each category (miRNA, Intergenic and All SNPs) over each of 1,000 equally-spaced bins spanning the range of negative log_10_(p-values) observed in the discovery group and for each of the 70 subdivisions. Every cumulative replication rate was calculated as the fraction of SNPs with a discovery negative log_10_(p-value) greater than the lower bound of the bin, that had a replication p-value smaller than 0.05. Average cumulative replication rates were subsequently computed across the 70 subdivisions. The True Discovery Rate - TDR (1-FDR) computed from the GWAS summary statistics can be thought of as the replication rate in an independent sample as the size of the replication sample goes to infinity. In practice, both the TDR and the cumulative replication rates are estimated with some error so that the former will not perfectly predict the latter. A substantial correspondence between the two is nonetheless to be expected for reasonable discovery and replication sample sizes.

## Results

### Enrichment of miRNA tagging SNPs

**Figure 1:**
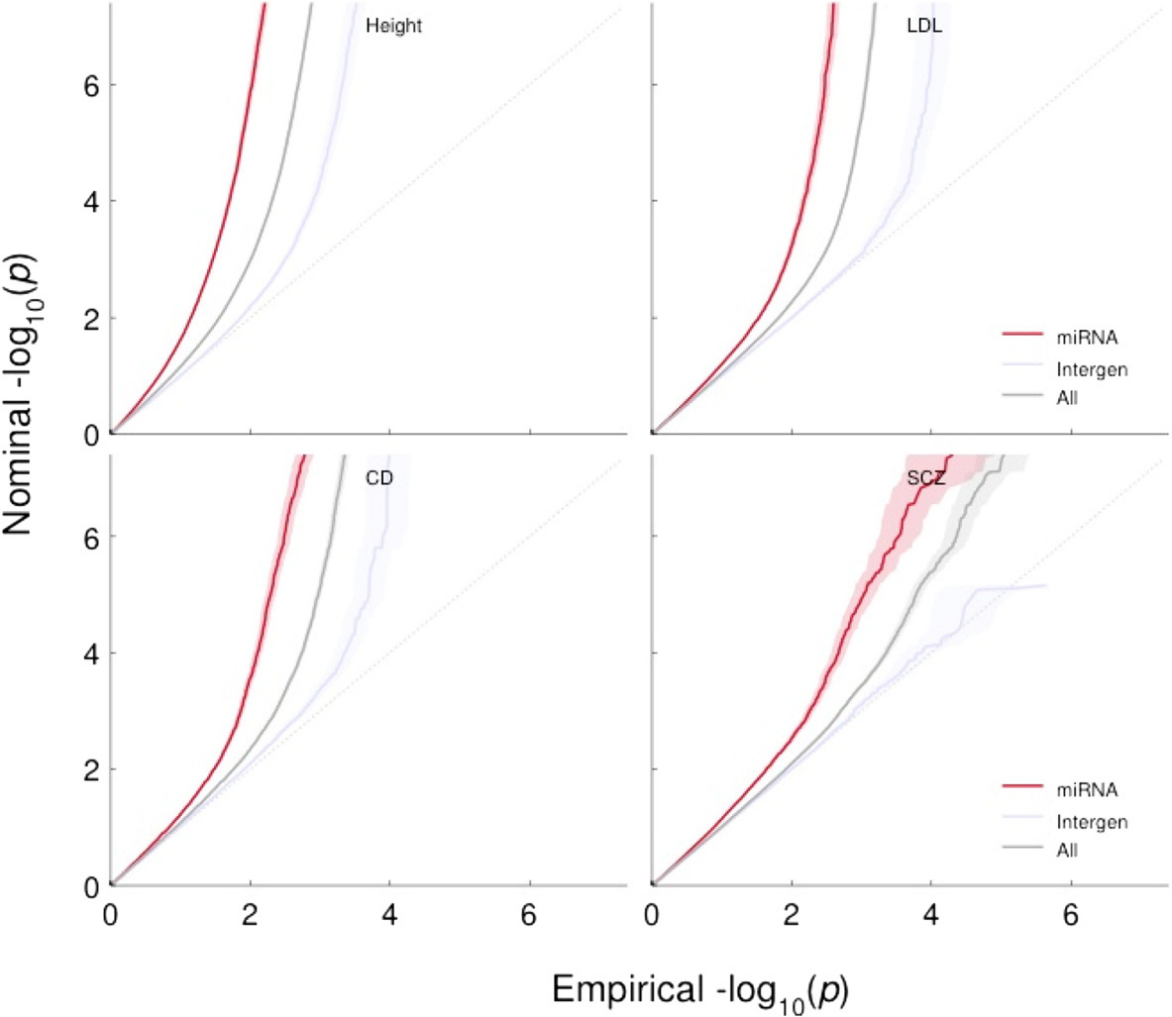
miRNA stratified Q-Q plots for Height, Low Density Lipoprotein (LDL), Crohn’s Disease (CD) and Schizophrenia (SCZ) using Linkage-Disequilibrium (LD)-weighted annotation scores. Shown are Q-Q plots for miRNA SNPs compared to those for all SNPs and intergenic SNPs, a collection of likely null SNPs. The confidence intervals were obtained by sampling ten independent sets of SNP representatives from all LD-blocks (r2 > 0.2) and computing means and confidence intervals for one thousand bins of nominal p-value.

The stratified Q-Q plots for height, LDL, CD and SCZ plotted with confidence intervals show a general enrichment pattern for miRNA (Figure 1) and miRNA-BS (Additional file 1) categories, illustrated by an early departure from the null line which is consistent with a greater proportion of true associations for a given p-value threshold. The deviation from the null line is more pronounced for miRNA and miRNA-BS–related SNPs than for all SNPs together or intergenic SNPs. In fact, miRNA and miRNA-BS seem largely as enriched as 3'UTR, among the annotation categories shown by Schork et al. [7] to have the largest abundance of associations. For example, the proportion of SNPs reaching a significance level of *p* < 10^−4^ is roughly ten times greater in the miRNA category than in the intergenic category. The same pattern is present in most of the other phenotypes, exception being CPD (Additional file 1). The parallel shapes of these curves are likely caused by the significant, though not complete, correlation among the categories due to the non-exclusive nature of the annotation scoring. Although the enrichment pattern of miRNAs is persistent through most of the phenotypes in the study, the shape of the curves varies across them.

This is consistent with different levels of polygenicity in different phenotypes but may also be due to different degrees of involvement of miRNA in the etiology of different phenotypes.

### Significance of enrichment

We computed significance values for the enrichment of each annotation category relative to intergenic SNPs, using the binomial proportion test (Table 1). The enrichment for miRNAs is nominally significant in all phenotypes except CPD and PrCa, and, after correcting for multiple testing of 15 phenotypes, only BD, MS, T2D become unconvincing; miRNA-BSs are significantly enriched in BD, BMI, CD, HDL, Height, LDL, SBP, TG, UC, and WHR (Additional file 1), but the effect is less evident across several of them and only BMI, Height, and LDL remain significant after correcting for multiple testing of 15 phenotypes. The significance of the enrichment in Height, LDL, CD and SCZ using LD-pruned SNPs is also illustrated with the visual aid of confidence intervals in Figure 1 and Additional file 1.

**Table 1:** **Significance of miRNA enrichment**

**Phenotype**	**BPT p-value**
BD	2.4e-02^§^
BMI	1.0e-12*
CD	1.9e-06*
CPD	6.3e-01
HDL	2.2e-20*
Height	3.9e-90*
LDL	2.6e-17*
MS	3.3e-03^§^
PrCa	1.1e-01
SBP	9.8e-08*
SCZ	4.1e-07*
T2D	9.8e-03^§^
TG	1.5e-16*
UC	3.2e-06*
WHR	5.8e-06*

Significance of miRNA enrichment in 15 phenotypes. Reported are the binomial proportion test (BPT) p-values for miRNA target regions compared with intergenic SNPs for all phenotypes. CPD and PrCa do not reach nominal statistical significance; BD, MS, and T2D would not pass strict multiple testing criteria for 15 phenotypes – BD, Bipolar Disorder; BMI, Body Mass Index; CD, Crohn's disease; CPD, Cigarettes per Day; HDL, High density lipoprotein; LDL, Low density lipoprotein; MS, multiple sclerosis; PrCa, prostate cancer; SBP, systolic blood pressure; SCZ, Schizophrenia; T2D, Type 2 Diabetes; TG, triglycerides; UC, Ulcerative Colitis; WHR, Waist to Hip Ratio. ^§^ Nominally significant (not significant after controlling for multiple testing); * Significant.

Due to the considerable differences among the studies the enrichment p-values may not be directly comparable to one another but they remain nonetheless a reliable indicator of significance.

### miRNA enrichment in relationship to other genomic regions

It was previously shown across several traits that the summary statistics resulting from SNPs in LD with 5'UTR and 3'UTR show the largest abundance of associations, while those from SNPs in LD with intergenic regions show a depletion of associations relative to the baseline [6,7,36]. We compared the relative pattern of enrichment of miRNA, 5’UTR and 3'UTR in stratified Q-Q plots for height, LDL, CD and SCZ (Additional file 1). In all four phenotypes miRNA-tagging SNPs show similar levels of enrichment as those tagging 5’UTR and 3’UTR. In order to rule out the possibility that the correlation with other genic categories might drive the enrichment of miRNA, following Schork et al.’s procedure [7], we regressed the summary statistics *z*^2^ against the LD-informed annotation values for miRNA, miRNA-BS, 3’UTR, 5’UTR, Exon, Intron as well as Intergenic and total LD. The regression showed a significant residual independent effect of miRNA on the z^2^ (Additional file 1).

### Quantification of Enrichment

**Figure 2:**
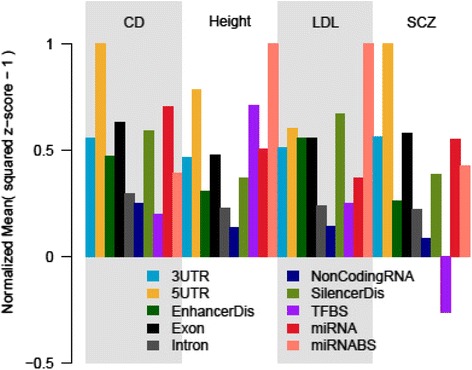
Categorical Enrichment for Height, Low Density Lipoprotein (LDL), Crohn’s Disease (CD) and Schizophrenia (SCZ). The relative pattern of enrichment of LD-weighted genic annotation categories, as measured by the mean (z-score^2^ - 1) normalized by the highest value across categories within each phenotype after intergenic inflation control, remains consistent. Results for all phenotypes are shown in Additional file 1.

The mean of *z*^2^-1 per SNP annotation category was used to get a quantitative measure of the enrichment of that category for association with each phenotype. The enrichment scores, normalized by the maximum value across categories within each phenotype, are presented in Figure 2 and Additional file 1. The 5′UTR annotation category shows the highest mean *z*^2^-1 in almost all phenotypes but is followed, often closely, by miRNA or miRNA-BS (Additional file 1).

### Replication rate and condFDR

**Figure 3:**
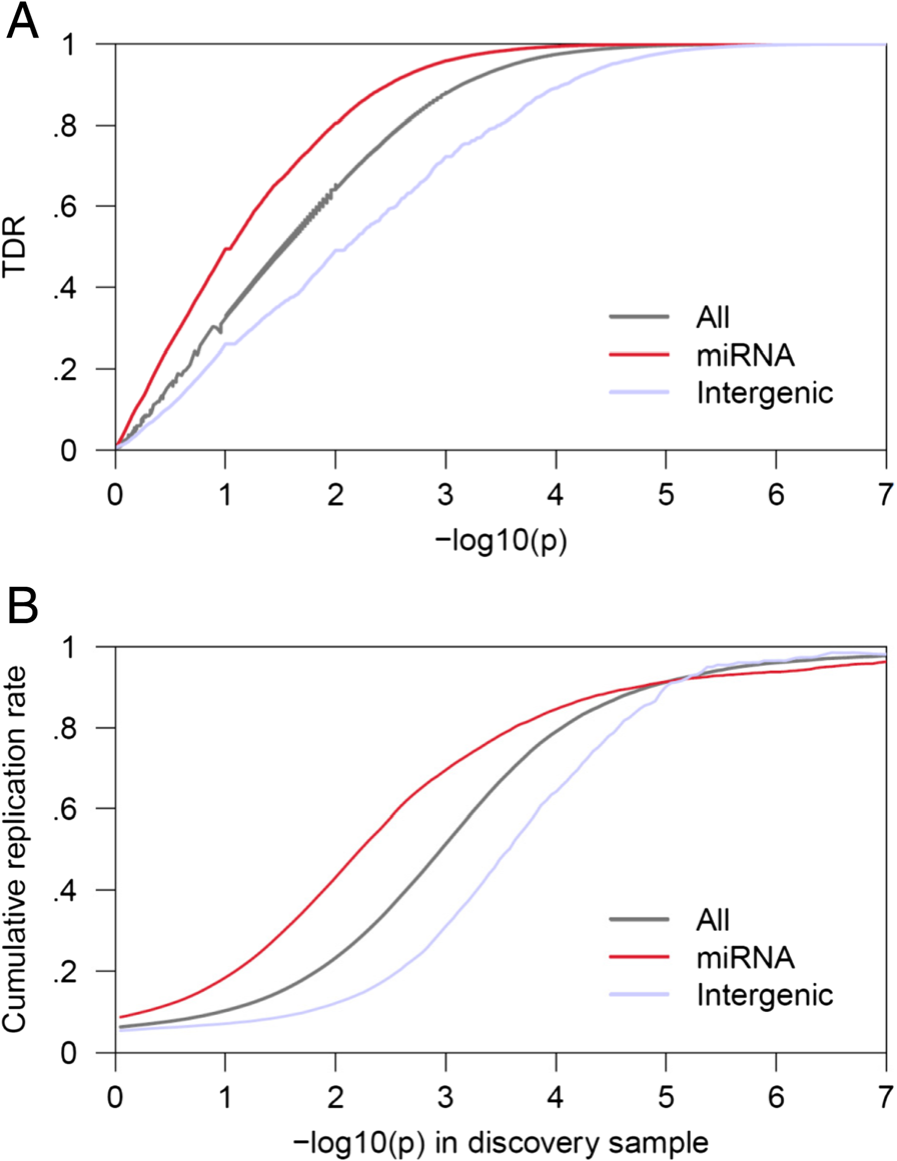
Independent study replication confirms enrichment in Crohn’s Disease. Shown are plots of **(A)** stratified true discovery rate (TDR) and **(B)** cumulative replication rate as functions of the negative decadic logarithm of the p-value. The p-value in the cumulative replication rate plot refers to the p-value in the discovery sample. Replication is intended as achieved for p-value smaller than 0.05 in the replication sample. It is evident from **(B)** how, given a p-value threshold, miRNA associations replicate at a higher rate in independent samples than just any SNPs or SNPs in intergenic regions. The vertical intercept is the overall replication rate per category. The cumulative replication rate mirrors the TDR as should be expected.

To further address the possibility that the observed pattern of differential enrichment results from spurious (i.e., non-generalizable) associations due to category-specific confounding effects or statistical modeling errors, we also studied the empirical replication rate across independent sub-studies of Crohn’s Disease (CD) for which eight sub-studies summary statistics were readily available. Figure 3 shows the empirical cumulative replication rate plots as a function of nominal p-value for miRNA, intergenic and all SNPs. As can be seen, an order of magnitude lower p-values are necessary for intergenic SNPs compared to miRNA SNPs s to achieve a wide range of replication rates (0.2 – 0.8). Or, seen from a different perspective, miRNA SNPs replicate at consistently higher rates, e.g. up to five times higher than intergenic SNPs for p-values lower than 0.01. This encouraged us to examine the conditional false discovery rates to assess the improvement achievable in the detection of miRNA-related loci. The condFDR method improves the discovery of SNPs in the miRNA category across all phenotypes, with an average ~ three-fold increase compared to standard methods (Table 2). Since this is accompanied by the same or a better replication rate, the method provides increased power.

**Table 2:** **Number of significant miRNA genomic loci**

**Phenotype**	**condFDR**	**FDR**	**p-value**
	**(<0.01)**	**(<0.01)**	**(Bonferroni)**
BD	8	2	0
BMI	7	5	3
CD	55	34	16
CPD	1	0	0
HDL	52	38	18
Height	189	123	51
LDL	62	37	19
MS	51	42	25
PrCa	16	12	6
SBP	12	6	2
SCZ	29	14	6
T2D	1	1	1
TG	84	43	20
UC	54	36	18
WHR	4	3	0

Comparative table showing number of identified genomic loci with conditional false discovery rate (condFDR < 0.01) compared with false discovery rate (FDR < 0.01) and standard p-value (Bonferroni correction p < 5×10^−8^). We show that the condFDR method improves the discovery of miRNA SNPs across the 15 phenotypes. The numbers reported here are after pruning SNPs for LD at a threshold of r^2^ ≤ 0.2. BD, Bipolar Disorder; BMI, Body Mass Index; CD, Crohn's disease; CPD, Cigarettes per Day; HDL, High density lipoprotein; LDL, Low density lipoprotein; MS, multiple sclerosis; PrCa, prostate cancer; SBP, systolic blood pressure; SCZ, Schizophrenia; T2D, Type 2 Diabetes; TG, triglycerides; UC, Ulcerative Colitis; WHR, Waist to Hip Ratio.

## Discussion

The main finding of the current study is that SNPs in miRNA-related regions show a significant and persistent pattern of association enrichment in a variety of complex human phenotypes. This supports the thesis that miRNAs play an important role in the etiology of these phenotypes, and further illustrates the polygenic architecture of complex human phenotypes.

The traditional statistical approach to GWASs has thus far only explained a small proportion of the observed heritability of complex human traits and disorders. However, previous studies have suggested that higher proportions of polygenic effects are to some extent actually detectable in existing GWAS data [37]. The statistical framework adopted here is based on the reasonable assumption that SNPs in different genomic regions are not equally likely to affect the phenotypes. Rather, as has been shown before, SNPs within certain functional regions of the genome [7] or SNPs with possible pleiotropic effects are more prone to show association.

A large share of miRNA transcription regions and, by their very nature, all miRNA target regions are clustered in or around genes. This exposes the SNPs held within such regions to correlation with SNPs in other genic categories (e.g., 3’UTR, 5’UTR, exons, introns). This correlation is further exasperated by the LD-weighting procedure applied to account for indirect effects. And indeed, controlling for this correlation does reduce the significance of miRNA enrichment, which however for the most part holds, suggesting it contains a residual pure miRNA component [38]. Further, the replication analysis shows clearly that SNPs tagging miRNA are more likely to be replicated, which is a very compelling argument in favour of true discoveries. The replication results emboldened us to use the LD-weighted miRNA annotations, combined with an established method for computing conditional False Discovery Rates, to improve the discovery of SNPs in GWAS.

The statistical framework employed here has elsewhere been exploited to identify specific SNP associations, which are otherwise undetected by traditional GWAS analyses [8,9]. The current findings suggest that leveraging information about miRNA affiliation leads to an increased number of identified gene loci, as shown for all phenotypes in Table 2. The increase is not due merely to an effective lowering of the significance threshold, since the condFDR method also re-ranks the SNPs, privileging those with higher prior likelihood of association. Indeed, for Crohn’s disease (Figure 3) we show that miRNA SNPs identified with the condFDR method have a higher probability of replicating in independent samples (replication rate). This is not the case for discoveries attained by lowering the threshold. Unfortunately, we did not have access to sub-studies from other meta-analyses, but we expect we would achieve a similar increase in statistical power for other complex phenotypes as well.

Induction or repression of miRNA expression can significantly influence most biological processes, such as apoptosis, cell fate specification, cell proliferation, DNA repair, cell cycle and DNA methylation. Associations between miRNAs and human diseases such as autoimmune diseases, skin diseases, neurological and psychiatric diseases, cancer, cardiovascular diseases, asthma and others have repeatedly confirmed the important biological role played by miRNA [39-43].

Disease-associated SNPs can affect miRNA in many ways e.g. altering its processing, maturation and expression. SNPs in both miRNAs and miRNA targets can then affect their interaction and binding affinity [17]. Indeed, it has been experimentally validated that 52% of SNPs in the dbSNP database would be able to create novel miRNA binding sites [44].

The first GWAS finding that was explained by polymorphic miRNA targeting was the synonymous SNP (c.313C > T) in the 3′UTR of *IRGM* (immunity-related GTPase family M protein) [45].This SNP decreases the binding of *mir-196* and is associated with the risk of Crohn's disease*.* Another SNP, rs1625579, located in the intron of a putative primary transcript for the *mir-137* gene, has been associated with schizophrenia [19], This SNP alters the seed sequence of miR-137 that is involved in neuronal development. Four other genes associated with schizophrenia (*TCF4* (transcription factor 4), *CACNA1C* (calcium channel, voltage-dependent, L type, alpha 1C subunit), *CSMD1* (CUB and Sushi multiple domains 1) and *C10orf26* (chromosome 10 open reading frame 26) contain predicted target-binding sites for miR-137, what indicates that the expression levels of these genes could also be affected by the mechanisms described above [17,19]).

The same miRNA can bind to several different mRNAs, and each mRNA can be bound by different miRNAs, thus their overall effect can be enhanced. Our study is not without limitations and these are largely due to weaknesses of the current miRNA target prediction methods. The mRNA targets of miRNAs can be predicted by bioinformatic algorithms such as PicTar, miRanda and TargetScan [46-48]. However, the prediction of the functional consequences of coding changes remains a challenge, since the majority of the data are derived from microarray analysis, with limitations of miRNA expression profile of the microarray chip. Relevant techniques such as deep sequencing, custom SNP arrays, as well as target capture methods and functional prediction efforts in complex traits (target-prediction algorithms) are rapidly emerging, bringing new hopes of better understanding to be achieved. Examining miRNAs profiles in tissues cells could further elucidate their involvement in complex pathologies. Since expression of miRNAs is influenced by environmental factors, the miRNA enrichment could help separating environmental and genetic factors involved in the development of disorders.

## Conclusions

In conclusion, the current findings demonstrate the enrichment of microRNA-related SNPs in a variety of GWASs of complex human phenotypes. Such enrichment can be used to improve the detection of significant associations and thus open new ways to further investigate mechanistic relationships between miRNAs and complex disorders.

## Additional file

Additional file 1:
**The PRACTICAL Consortiumin.**
