# Supplementary material for: MicroRNAs enrichment in GWAS of complex human phenotypes
Source: BMC Genomics. 2015 Apr 16;16(1):304. doi: 10.1186/s12864-015-1513-5 (PMC4437677; doi:10.1186/s12864-015-1513-5)
Supplement: Additional file 1: — The PRACTICAL Consortiumin. [file 12864_2015_1513_MOESM1_ESM.doc]

**SUPPLEMENTARY MATERIAL**

*MicroRNAs Enrichment in GWAS of Complex Human Phenotypes.*

Luiz F. Goulart, Francesco Bettella, Ida E. Sønderby, Andrew J. Schork, Wesley K. Thompson, Morten Mattingsdal, Vidar M. Steen, Verena Zuber, Yunpeng Wang, Anders M. Dale, PRACTICAL/ELLIPSE consortium, Ole A. Andreassen, and Srdjan Djurovic

**Figure S1.**

**Figure S1. miRNA-BS stratified Q-Q plots for Height, Low Density Lipoprotein (LDL), Crohn’s Disease (CD) and Schizophrenia (SCZ). Shown are Q-Q plots for miRNA-BS SNPs compared to those for all SNPs and intergenic SNPs, a collection of likely null SNPs. The confidence intervals were obtained by sampling ten independent sets of SNP representatives from all LD-blocks (*r*^2^>0.2) and computing means and confidence intervals for one thousand bins of nominal p-value.**

**Figure S2.**

**Figure S2. Stratified Q-Q plots for Height, Low Density Lipoprotein (LDL), Crohn’s Disease (CD) and Schizophrenia (SCZ). The plots show different enrichment patterns for different (LD)-weighted annotation categories (miRNA, 5’UTR, 3’UTR).** In particular, 5’UTR confirms to generally be the most enriched genic category but miRNA mostly follows similar patterns. Observing the high level of parallelism among the curves, the suspicion of high correlation between the categories can arise. This was settled by performing a linear regression of the *z*-scores against a number of potentially correlated categorical annotation scores (SupplementaryTable 2).

**Figure S3.**

**Figure S3. Categorical enrichment for 11 phenotypes.** The relative pattern of enrichment of LD-weighted genic annotation categories (3’UTR, 5’UTR, enhancer disruptor (EnhancerDis), exon, intron, non-coding RNA, silencer disruptor (SilencerDis), transcription factor binding site (TFBS), miRNA transcription (miRNA), miRNA binding sites (miRNA-BS)), as measured by the mean (z-score^2^ - 1) normalized by the highest value across categories within each phenotype after intergenic inflation control, remains consistent. Bipolar Disorder (BD); Body Mass Index (BMI); Cigarettes per Day (CPD); High Density Lipoprotein (HDL); Multiple Sclerosis (MS); Prostate Cancer (PrCa); Systolic Blood Pressure (SBP); Type 2 diabetes (T2D); Triglycerides (TG); Ulcerative Colitis (UC); Waist to Hip Ratio (WHR).

**Figure S4.**

**Figure S4. miRNA stratified Q-Q plots across 11 phenotypes. Similar miRNA enrichment patterns are discernible across a wide variety of other phenotypes, the only exceptions being CPD and PrCa.** Bipolar Disorder (BD); Body Mass Index (BMI); Cigarettes per Day (CPD); High Density Lipoprotein (HDL); Multiple Sclerosis (MS); Prostate Cancer (PrCa); Systolic Blood Pressure (SBP); Type 2 diabetes (T2D); Triglycerides (TG); Ulcerative Colitis (UC); Waist to Hip Ratio (WHR).

**Table S1 : Description of the underlying GWAS data**

| Disorder group | Trait | N | #SNPs | Minimum p-value | GWS SNPs |
| --- | --- | --- | --- | --- | --- |
| Brain-related | BD | 16,731 | 2,381,661 | 5.54 e-10 | 42 |
|  | SCZ | 21,856 | 1,171,056 | 4.30 e-11 | 101 |
|  | CPD | 74,053 | 2,397,337 | 4.23 e-35 | 128 |
| Cardiovascular and Metabolic | SBP | 203,056 | 2,382,073 | 9.73 e-13 | 107 |
|  | HDL | 96,598 | 2,508,370 | 1.98 e-323 | 2,165 |
|  | LDL | 99,900 | 2,508,375 | 9.70 e-171 | 1,704 |
|  | TG | 96,568 | 2,508,363 | 6.71 e-240 | 1,706 |
|  | T2D | 141,454 | 2,426,886 | 5.0 e-8 | 12 |
| Immune Mediated | MS | 27,148 | 441,547 | 1.0 e-12 | 29 |
|  | CD | 51,109 | 942,858 | 4 .0e-69 | 968 |
|  | UC | 26,405 | 1,273,589 | 4.62 e-77 | 671 |
| Anthropomorphic Measures | BMI | 123,865 | 2,400,377 | 2.05 e-62 | 765 |
|  | Height | 183,727 | 2,398,527 | 4.47 e-52 | 4,456 |
|  | WHR | 77,167 | 2,376,820 | 7.66 e-15 | 296 |
| Cancer | PrCa | 25,074 | 211,155 | 5.0 e-8 | 23 |

***References :***

Bipolar disorder (BD)

schizophrenia (SCZ)

smoking behaviour as measured by cigarettes per day (CPD)

systolic blood pressure (SBP)

plasma lipids (triglycerides, TG[22]

high density lipoprotein, HDL[22]

low density lipoprotein, LDL)

type 2 diabetes

multiple sclerosis (MS)

Crohn’s disease (CD)

ulcerative colitis (UC)

body mass index (BMI)

height

waist to hip ratio (WHR)

prostate cancer

**Table S2: Significance of miRNA-BS enrichment.**

| Phenotype | BPT p-value |
| --- | --- |
| BD | 2.4e-03**^§^** |
| BMI | 8.2e-04* |
| CD | 2.5e-02**^§^** |
| CPD | 7.6e-01 |
| HDL | 3.1e-03**^§^** |
| Height | 5.4e-39* |
| LDL | 1.5e-04* |
| MS | 5.2e-01 |
| PrCa | 5.1e-02 |
| SBP | 7.9e-03**^§^** |
| SCZ | 1.8e-01 |
| T2D | 1.5e-01 |
| TG | 2.8e-03**^§^** |
| UC | 5.8e-03**^§^** |
| WHR | 1.6e-02**^§^** |

**Table S2: Significance of miRNA binding sites (miRNA-BS) enrichment in all phenotypes.** Reported are the binomial proportion test p-values for miRNA binding sites compared with intergenic SNPs for all phenotypes. CPD, MS, PrCa, SCZ and T2D do not reach nominal statistical significance; BD, CD, HDL, SBP, TG, UC, and WHR would not pass strict multiple testing criteria for 15 phenotypes – BD, Bipolar Disorder; BMI, Body Mass Index; CD, Crohn's disease; CPD, Cigarettes per Day; HDL, High density lipoprotein; LDL, Low density lipoprotein; MS, multiple sclerosis; SBP, systolic blood pressure; SCZ, Schizophrenia; TG, triglycerides; UC, Ulcerative Colitis, WHR Waist to Hip Ratio. **§** Nominally significant (not significant after controlling for multiple testing); * Significant.

**Table S3. Regression analysis of all 15 phenotypes. The effects of miRNA and miRNA-BS LD-weighted annotation scores** on the z-score remains mostly significant upon controlling for the other categorical annotation scores and the total amount of LD.

| Bipolar disorder | | | |
| --- | --- | --- | --- |
| variable | coeff. | SE | p-value |
| miRNA | 0.0014 | 0.0004 | 1.4e-04 |
| miRNABS | -0.0096 | 0.0054 | 7.9e-02 |
| Exon | -0.0180 | 0.0029 | 3.1e-10 |
| Intron | 0.0001 | 0.0000 | 1.3e-05 |
| 3UTR | 0.0048 | 0.0012 | 6.2e-05 |
| 5UTR | 0.0234 | 0.0035 | 3.0e-11 |
| EnhancerDis | 0.0309 | 0.0033 | 1.8e-20 |
| SilencerDis | -0.0043 | 0.0039 | 2.7e-01 |
| NonCodingRNA | -0.0006 | 0.0001 | 2.3e-18 |
| TFBS | -0.0005 | 0.0153 | 9.7e-01 |
| Intergenic | -0.0509 | 0.0039 | 2.6e-38 |
| TotLD | 0.0005 | 0.0000 | 5.9e-152 |
|  |  |  |  |
| Body mass index | | | |
| variable | coeff. | SE | p-value |
| miRNA | 0.0050 | 0.0007 | 2.6e-13 |
| miRNABS | 0.0497 | 0.0102 | 1.2e-06 |
| Exon | -0.0095 | 0.0054 | 7.7e-02 |
| Intron | 0.0010 | 0.0001 | 1.6e-59 |
| 3UTR | 0.0229 | 0.0022 | 1.2e-24 |
| 5UTR | 0.0445 | 0.0066 | 1.6e-11 |
| EnhancerDis | -0.0055 | 0.0063 | 3.8e-01 |
| SilencerDis | -0.0316 | 0.0073 | 1.3e-05 |
| NonCodingRNA | 0.0004 | 0.0001 | 1.5e-03 |
| TFBS | -0.2550 | 0.0288 | 9.4e-19 |
| Intergenic | 0.0075 | 0.0074 | 3.1e-01 |
| TotLD | 0.0008 | 0.0000 | 6.2e-95 |
|  |  |  |  |
| Crohn's disease | | | |
| variable | coeff. | SE | p-value |
| miRNA | 0.0073 | 0.0028 | 1.0e-02 |
| miRNABS | 0.0026 | 0.0408 | 9.5e-01 |
| Exon | 0.0511 | 0.0219 | 1.9e-02 |
| Intron | 0.0003 | 0.0003 | 2.5e-01 |
| 3UTR | 0.0358 | 0.0092 | 9.3e-05 |
| 5UTR | 0.0556 | 0.0271 | 4.0e-02 |
| EnhancerDis | -0.0010 | 0.0256 | 9.7e-01 |
| SilencerDis | -0.0152 | 0.0307 | 6.2e-01 |
| NonCodingRNA | 0.0006 | 0.0006 | 3.3e-01 |
| TFBS | -0.1901 | 0.1299 | 1.4e-01 |
| Intergenic | -0.0638 | 0.0331 | 5.4e-02 |
| TotLD | 0.0002 | 0.0002 | 1.8e-01 |
|  |  |  |  |
| Cigarettes per day | | | |
| variable | coeff. | SE | p-value |
| miRNA | -0.0003 | 0.0004 | 4.9e-01 |
| miRNABS | -0.0150 | 0.0060 | 1.3e-02 |
| Exon | 0.0086 | 0.0032 | 6.4e-03 |
| Intron | -0.0005 | 0.0000 | 2.0e-42 |
| 3UTR | -0.0011 | 0.0013 | 4.0e-01 |
| 5UTR | -0.0095 | 0.0039 | 1.5e-02 |
| EnhancerDis | -0.0138 | 0.0037 | 1.8e-04 |
| SilencerDis | 0.0244 | 0.0043 | 1.2e-08 |
| NonCodingRNA | -0.0008 | 0.0001 | 1.1e-24 |
| TFBS | 0.0055 | 0.0170 | 7.4e-01 |
| Intergenic | 0.0189 | 0.0044 | 1.5e-05 |
| TotLD | 0.0004 | 0.0000 | 1.6e-92 |
|  |  |  |  |
| High density lipoprotein | | | |
| variable | coeff. | SE | p-value |
| miRNA | 0.0037 | 0.0004 | 1.5e-21 |
| miRNABS | 0.0188 | 0.0059 | 1.5e-03 |
| Exon | 0.0227 | 0.0031 | 2.3e-13 |
| Intron | 0.0002 | 0.0000 | 3.3e-10 |
| 3UTR | 0.0325 | 0.0013 | 1.6e-139 |
| 5UTR | 0.0646 | 0.0038 | 3.4e-64 |
| EnhancerDis | -0.0065 | 0.0036 | 7.1e-02 |
| SilencerDis | -0.0139 | 0.0042 | 9.1e-04 |
| NonCodingRNA | -0.0001 | 0.0001 | 2.1e-01 |
| TFBS | -0.1877 | 0.0167 | 2.5e-29 |
| Intergenic | -0.0406 | 0.0043 | 1.6e-21 |
| TotLD | 0.0003 | 0.0000 | 2.0e-36 |
|  |  |  |  |
| Height | | | |
| variable | coeff. | SE | p-value |
| miRNA | 0.0058 | 0.0021 | 6.7e-03 |
| miRNABS | 0.2862 | 0.0324 | 9.2e-19 |
| Exon | 0.0075 | 0.0169 | 6.6e-01 |
| Intron | 0.0006 | 0.0002 | 3.7e-03 |
| 3UTR | 0.0457 | 0.0071 | 9.6e-11 |
| 5UTR | 0.2024 | 0.0209 | 3.2e-22 |
| EnhancerDis | 0.0366 | 0.0198 | 6.4e-02 |
| SilencerDis | -0.0088 | 0.0229 | 7.0e-01 |
| NonCodingRNA | 0.0006 | 0.0004 | 1.2e-01 |
| TFBS | 0.0574 | 0.0911 | 5.3e-01 |
| Intergenic | -0.3742 | 0.0234 | 8.6e-58 |
| TotLD | 0.0003 | 0.0001 | 1.6e-02 |
|  |  |  |  |
| Low density lipoprotein | | | |
| variable | coeff. | SE | p-value |
| miRNA | 0.0028 | 0.0004 | 8.1e-13 |
| miRNABS | 0.0872 | 0.0060 | 4.5e-48 |
| Exon | -0.0251 | 0.0031 | 8.9e-16 |
| Intron | 0.0009 | 0.0000 | 1.0e-131 |
| 3UTR | 0.0201 | 0.0013 | 1.3e-53 |
| 5UTR | 0.0782 | 0.0039 | 2.6e-91 |
| EnhancerDis | 0.0582 | 0.0036 | 1.7e-57 |
| SilencerDis | -0.0540 | 0.0042 | 2.8e-37 |
| NonCodingRNA | 0.0006 | 0.0001 | 1.1e-15 |
| TFBS | -0.0472 | 0.0169 | 5.1e-03 |
| Intergenic | -0.0408 | 0.0043 | 2.3e-21 |
| TotLD | 0.0000 | 0.0000 | 5.7e-01 |
|  |  |  |  |
| Multiple sclerosis | | | |
| variable | coeff. | SE | p-value |
| miRNA | 0.0019 | 0.0010 | 6.8e-02 |
| miRNABS | 0.0394 | 0.0162 | 1.5e-02 |
| Exon | 0.0057 | 0.0087 | 5.1e-01 |
| Intron | 0.0000 | 0.0001 | 9.7e-01 |
| 3UTR | 0.0261 | 0.0038 | 4.9e-12 |
| 5UTR | 0.0550 | 0.0111 | 6.6e-07 |
| EnhancerDis | 0.0530 | 0.0103 | 2.8e-07 |
| SilencerDis | -0.0483 | 0.0119 | 4.8e-05 |
| NonCodingRNA | 0.0009 | 0.0002 | 1.8e-04 |
| TFBS | 0.0484 | 0.0515 | 3.5e-01 |
| Intergenic | -0.0452 | 0.0110 | 3.9e-05 |
| TotLD | -0.0001 | 0.0001 | 1.4e-01 |
|  |  |  |  |
| Prostate Cancer | | | |
| variable | coeff. | SE | p-value |
| miRNA | 0.0011 | 0.0015 | 4.7e-01 |
| miRNABS | 0.0332 | 0.0169 | 5.0e-02 |
| Exon | 0.0218 | 0.0127 | 8.7e-02 |
| Intron | 0.0006 | 0.0002 | 3.3e-04 |
| 3UTR | 0.0084 | 0.0050 | 9.5e-02 |
| 5UTR | 0.0811 | 0.0149 | 4.8e-08 |
| EnhancerDis | -0.0039 | 0.0148 | 7.9e-01 |
| SilencerDis | 0.0495 | 0.0179 | 5.7e-03 |
| NonCodingRNA | 0.0045 | 0.0003 | 5.3e-39 |
| TFBS | -0.2391 | 0.0682 | 4.6e-04 |
| Intergenic | -0.0752 | 0.0158 | 1.8e-06 |
| TotLD | 0.0004 | 0.0001 | 2.9e-05 |
|  |  |  |  |
| Systolic blood pressure | | | |
| variable | coeff. | SE | p-value |
| miRNA | 0.0043 | 0.0007 | 2.1e-10 |
| miRNABS | -0.0656 | 0.0103 | 2.2e-10 |
| Exon | -0.0209 | 0.0054 | 1.1e-04 |
| Intron | 0.0006 | 0.0001 | 1.0e-22 |
| 3UTR | 0.0223 | 0.0022 | 3.1e-23 |
| 5UTR | 0.0095 | 0.0066 | 1.5e-01 |
| EnhancerDis | 0.0605 | 0.0063 | 8.9e-22 |
| SilencerDis | -0.0426 | 0.0073 | 5.5e-09 |
| NonCodingRNA | -0.0007 | 0.0001 | 2.5e-07 |
| TFBS | 0.0592 | 0.0287 | 3.9e-02 |
| Intergenic | -0.0461 | 0.0073 | 2.9e-10 |
| TotLD | 0.0001 | 0.0000 | 9.6e-05 |
|  |  |  |  |
| Schizophrenia | | | |
| variable | coeff. | SE | p-value |
| miRNA | 0.0020 | 0.0005 | 4.7e-05 |
| miRNABS | 0.0238 | 0.0074 | 1.4e-03 |
| Exon | 0.0044 | 0.0039 | 2.6e-01 |
| Intron | 0.0003 | 0.0001 | 6.1e-11 |
| 3UTR | 0.0199 | 0.0017 | 4.1e-33 |
| 5UTR | 0.0752 | 0.0048 | 4.9e-55 |
| EnhancerDis | 0.0230 | 0.0045 | 3.2e-07 |
| SilencerDis | -0.0796 | 0.0055 | 2.0e-47 |
| NonCodingRNA | -0.0005 | 0.0001 | 3.4e-05 |
| TFBS | -0.1728 | 0.0237 | 2.9e-13 |
| Intergenic | -0.0396 | 0.0058 | 1.3e-11 |
| TotLD | 0.0009 | 0.0000 | 1.9e-186 |
|  |  |  |  |
| Type-2 diabetes | | | |
| variable | coeff. | SE | p-value |
| miRNA | 0.0041 | 0.0007 | 1.1e-08 |
| miRNABS | 0.0700 | 0.0107 | 5.1e-11 |
| Exon | -0.0236 | 0.0056 | 2.7e-05 |
| Intron | 0.0003 | 0.0001 | 7.1e-07 |
| 3UTR | 0.0029 | 0.0023 | 2.1e-01 |
| 5UTR | 0.0159 | 0.0069 | 2.1e-02 |
| EnhancerDis | 0.0303 | 0.0066 | 4.0e-06 |
| SilencerDis | 0.0268 | 0.0076 | 4.4e-04 |
| NonCodingRNA | -0.0003 | 0.0001 | 1.4e-02 |
| TFBS | 0.2272 | 0.0298 | 2.3e-14 |
| Intergenic | -0.0146 | 0.0078 | 6.1e-02 |
| TotLD | 0.0001 | 0.0000 | 2.3e-02 |
|  |  |  |  |
| Triglycerides | | | |
| variable | coeff. | SE | p-value |
| miRNA | 0.0070 | 0.0004 | 4.3e-70 |
| miRNABS | 0.0520 | 0.0060 | 8.2e-18 |
| Exon | -0.0158 | 0.0032 | 5.5e-07 |
| Intron | 0.0007 | 0.0000 | 3.2e-92 |
| 3UTR | 0.0163 | 0.0013 | 6.2e-35 |
| 5UTR | 0.0806 | 0.0039 | 6.7e-95 |
| EnhancerDis | 0.0392 | 0.0037 | 2.1e-26 |
| SilencerDis | -0.0480 | 0.0043 | 3.4e-29 |
| NonCodingRNA | 0.0002 | 0.0001 | 1.1e-03 |
| TFBS | -0.1447 | 0.0170 | 2.0e-17 |
| Intergenic | -0.0180 | 0.0043 | 3.5e-05 |
| TotLD | 0.0001 | 0.0000 | 1.7e-08 |
|  |  |  |  |
| Ulcerative colitis | | | |
| variable | coeff. | SE | p-value |
| miRNA | 0.0030 | 0.0009 | 8.3e-04 |
| miRNABS | 0.0640 | 0.0131 | 1.1e-06 |
| Exon | 0.0183 | 0.0069 | 8.1e-03 |
| Intron | -0.0001 | 0.0001 | 5.7e-01 |
| 3UTR | 0.0224 | 0.0029 | 2.9e-14 |
| 5UTR | 0.0474 | 0.0086 | 3.1e-08 |
| EnhancerDis | 0.0208 | 0.0080 | 9.6e-03 |
| SilencerDis | -0.0404 | 0.0097 | 3.0e-05 |
| NonCodingRNA | 0.0000 | 0.0002 | 9.4e-01 |
| TFBS | -0.0055 | 0.0416 | 9.0e-01 |
| Intergenic | -0.0322 | 0.0102 | 1.6e-03 |
| TotLD | 0.0000 | 0.0001 | 4.8e-01 |
|  |  |  |  |
| Waist-Hip ratio | | | |
| variable | coeff. | SE | p-value |
| miRNA | 0.0008 | 0.0019 | 6.7e-01 |
| miRNABS | -0.0238 | 0.0287 | 4.1e-01 |
| Exon | 0.0370 | 0.0150 | 1.4e-02 |
| Intron | 0.0018 | 0.0002 | 8.2e-27 |
| 3UTR | 0.0116 | 0.0062 | 6.4e-02 |
| 5UTR | 0.0298 | 0.0185 | 1.1e-01 |
| EnhancerDis | -0.0144 | 0.0175 | 4.1e-01 |
| SilencerDis | -0.0463 | 0.0203 | 2.3e-02 |
| NonCodingRNA | 0.0010 | 0.0004 | 3.0e-03 |
| TFBS | 0.0947 | 0.0797 | 2.3e-01 |
| Intergenic | -0.0451 | 0.0204 | 2.7e-02 |
| TotLD | -0.0001 | 0.0001 | 3.4e-01 |

**Table S4. – Number of SNPs in each LD-informed genic category.**

| Genic Category | No. of SNPs |
| --- | --- |
| miRNA | 181933 |
| miRNA-BS | 16596 |
| 3UTR | 331312 |
| 5UTR | 98502 |
| Exon | 317087 |
| Intron | 1496208 |
| EnhancerDis | 267602 |
| SilencerDis | 165937 |
| NonCodingRNA | 490922 |
| TFBS | 4814 |
| Intergenic | 487225 |

**The PRACTICAL Consortium (http://practical.ccge.medschl.cam.ac.uk/):**

Rosalind Eeles ^1, 2^, Doug Easton ^3^, Zsofia Kote-Jarai ^1^, Ali Amin Al Olama ^3^, Sara Benlloch ^3^, Kenneth Muir ^4^, Graham G. Giles ^5, 6^, Fredrik Wiklund ^7^, Henrik Gronberg ^7^, Christopher A. Haiman ^8^, Johanna Schleutker ^9, 10^, Maren Weischer ^11^, Ruth C. Travis ^12^, David Neal ^13^, Paul Pharoah ^14^, Kay-Tee Khaw ^15^, Janet L. Stanford ^16, 17^, William J. Blot ^18^, Stephen Thibodeau ^19^, Christiane Maier ^20, 21^, Adam S. Kibel ^22, 23^, Cezary Cybulski ^24^, Lisa Cannon-Albright ^25^, Hermann Brenner ^26^, Jong Park ^27^, Radka Kaneva ^28^, Jyotsna Batra ^29^, Manuel R. Teixeira ^30^, Hardev Pandha^31^

^1^ The Institute of Cancer Research, 15 Cotswold Road, Sutton, Surrey, SM2 5NG, UK, ^2^ Royal Marsden NHS Foundation Trust, Fulham and Sutton, London and Surrey, UK, ^3^ Centre for Cancer Genetic Epidemiology, Department of Public Health and Primary Care, University of Cambridge, Strangeways Laboratory, Worts Causeway, Cambridge, UK, ^4^ University of Warwick, Coventry, UK, ^5^ Cancer Epidemiology Centre, Cancer Council Victoria, 615 St Kilda Road, Melbourne Victoria, Australia, ^6^ Centre for Epidemiology and Biostatistics, Melbourne School of Population and Global Health, The University of Melbourne, Victoria, Australia, ^7^ Department of Medical Epidemiology and Biostatistics, Karolinska Institute, Stockholm, Sweden, ^8^ Department of Preventive Medicine, Keck School of Medicine, University of Southern California/Norris Comprehensive Cancer Center, Los Angeles, California, USA, ^9^ Department of Medical Biochemistry and Genetics, University of Turku, Turku, Finland, ^10^ Institute of Biomedical Technology/BioMediTech, University of Tampere and FimLab Laboratories, Tampere, Finland, ^11^ Department of Clinical Biochemistry, Herlev Hospital, Copenhagen University Hospital, Herlev Ringvej 75, DK-2730 Herlev, Denmark, ^12^ Cancer Epidemiology Unit, Nuffield Department of Clinical Medicine, University of Oxford, Oxford, UK, ^13^ Surgical Oncology (Uro-Oncology: S4), University of Cambridge, Box 279, Addenbrooke’s Hospital, Hills Road, Cambridge, UK and Cancer Research UK Cambridge Research Institute, Li Ka Shing Centre, Cambridge, UK, ^14^ Centre for Cancer Genetic Epidemiology, Department of Oncology, University of Cambridge, Strangeways Laboratory, Worts Causeway, Cambridge, UK, ^15^ Cambridge Institute of Public Health, University of Cambridge, Forvie Site, Robinson Way, Cambridge CB2 0SR, ^16^ Division of Public Health Sciences, Fred Hutchinson Cancer Research Center, Seattle, Washington, USA, ^17^ Department of Epidemiology, School of Public Health, University of Washington, Seattle, Washington, USA, ^18^ International Epidemiology Institute, 1455 Research Blvd., Suite 550, Rockville, MD 20850, ^19^ Mayo Clinic, Rochester, Minnesota, USA, ^20^ Department of Urology, University Hospital Ulm, Germany, ^21^ Institute of Human Genetics University Hospital Ulm, Germany, ^22^ Brigham and Women's Hospital/Dana-Farber Cancer Institute, 45 Francis Street- ASB II-3, Boston, MA 02115, ^23^ Washington University, St Louis, Missouri, ^24^ International Hereditary Cancer Center, Department of Genetics and Pathology, Pomeranian Medical University, Szczecin, Poland, ^25^ Division of Genetic Epidemiology, Department of Medicine, University of Utah School of Medicine, ^26^ Division of Clinical Epidemiology and Aging Research, German Cancer Research Center, Heidelberg Germany, ^27^ Division of Cancer Prevention and Control, H. Lee Moffitt Cancer Center, 12902 Magnolia Dr., Tampa, Florida, USA, ^28^ Molecular Medicine Center and Department of Medical Chemistry and Biochemistry, Medical University - Sofia, 2 Zdrave St, 1431, Sofia, Bulgaria, ^29^ Australian Prostate Cancer Research Centre-Qld, Institute of Health and Biomedical Innovation and Schools of Life Science and Public Health, Queensland University of Technology, Brisbane, Australia, ^30^ Department of Genetics, Portuguese Oncology Institute, Porto, Portugal and Biomedical Sciences Institute (ICBAS), Porto University, Porto, Portugal, ^31^The University of Surrey, Guildford, Surrey, GU2 7XH

**COGS acknowledgement and funding:**

This study would not have been possible without the contributions of the following: Per Hall (COGS); Douglas F. Easton, Paul Pharoah, Kyriaki Michailidou, Manjeet K. Bolla, Qin Wang (BCAC), Andrew Berchuck (OCAC), Rosalind A. Eeles, Douglas F. Easton, Ali Amin Al Olama, Zsofia Kote-Jarai, Sara Benlloch (PRACTICAL), Georgia Chenevix-Trench, Antonis Antoniou, Lesley McGuffog, Fergus Couch and Ken Offit (CIMBA), Joe Dennis, Alison M. Dunning, Andrew Lee, and Ed Dicks, Craig Luccarini and the staff of the Centre for Genetic Epidemiology Laboratory, Javier Benitez, Anna Gonzalez-Neira and the staff of the CNIO genotyping unit, Jacques Simard and Daniel C. Tessier, Francois Bacot, Daniel Vincent, Sylvie LaBoissière and Frederic Robidoux and the staff of the McGill University and Génome Québec Innovation Centre, Stig E. Bojesen, Sune F. Nielsen, Borge G. Nordestgaard, and the staff of the Copenhagen DNA laboratory, and Julie M. Cunningham, Sharon A. Windebank, Christopher A. Hilker, Jeffrey Meyer and the staff of Mayo Clinic Genotyping Core Facility

Funding for the iCOGS infrastructure came from: the European Community's Seventh Framework Programme under grant agreement n° 223175 (HEALTH-F2-2009-223175) (COGS), Cancer Research UK (C1287/A10118, C1287/A 10710, C12292/A11174, C1281/A12014, C5047/A8384, C5047/A15007, C5047/A10692), the National Institutes of Health (CA128978) and Post-Cancer GWAS initiative (1U19 CA148537, 1U19 CA148065 and 1U19 CA148112 - the GAME-ON initiative), the Department of Defence (W81XWH-10-1-0341), the Canadian Institutes of Health Research (CIHR) for the CIHR Team in Familial Risks of Breast Cancer, Komen Foundation for the Cure, the Breast Cancer Research Foundation, and the Ovarian Cancer Research Fund.
